# Metabolite Profiling of *Alocasia gigantea* Leaf Extract and Its Potential Anticancer Effect through Autophagy in Hepatocellular Carcinoma

**DOI:** 10.3390/molecules27238504

**Published:** 2022-12-03

**Authors:** Hend Okasha, Tarek Aboushousha, Manuel A. Coimbra, Susana M. Cardoso, Mosad A. Ghareeb

**Affiliations:** 1 Department of Biochemistry and Molecular Biology, Theodor Bilharz Research Institute, Kornaish El Nile, Warrak El-Hadar, Imbaba, P.O. Box 30, Giza 12411, Egypt; 2 Department of Pathology, Theodor Bilharz Research Institute, Kornaish El Nile, Warrak El-Hadar, Imbaba, P.O. Box 30, Giza 12411, Egypt; 3 LAQV-REQUIMTE, Department of Chemistry, University of Aveiro, 3810-193 Aveiro, Portugal; 4 Department of Medicinal Chemistry, Theodor Bilharz Research Institute, Kornaish El Nile, Warrak El-Hadar, Imbaba, P.O. Box 30, Giza 12411, Egypt

**Keywords:** *Alocasia gigantea*, hepatocellular carcinoma, autophagy, histopathology, UHPLC–DAD–ESI–MS/MS, phenolic compounds

## Abstract

Hepatocellular carcinoma (HCC) is a poor-prognosis type of cancer with high resistance to chemotherapy, making the search for safe drugs a mandatory issue. Plant-derived products have potential to reduce negative side effects of cancer treatments. In this work, ability of a defatted methanolic extract of *Alocasia gigantea* leaves to fight HCC was evaluated in an animal model. Overall, treatment of HCC-induced mice with the methanolic extract at 150 mg/kg body weight for four consecutive weeks caused induction of autophagy through silencing of the relative expression of autophagy suppressor (mTOR) and inducement of autophagy markers (AMPK, Beclin-1, and LC-3). Moreover, it improved preservation of the hepatic histological architecture of the animals, with minor hepatocytic changes but scattered foci of hepatocytic apoptosis. Chemical profiling of the methanolic extract via ultra-high-performance liquid chromatography coupled to a diode array detector and an electrospray mass spectrometer (UHPLC–DAD–ESI–MS/MS) allowed identification of di-*C*-glycosyl flavones, mostly represented by 6-*C*-hexosyl-8-*C*-pentosyl apigenin isomers, which may possibly be associated with inducement of the autophagy pathway in HCC. Overall, these outcomes gave an initial visualization of the operative effect of some compounds in *A. gigantea* leaves that are potential treatment for HCC.

## 1. Introduction

Hepatocellular carcinoma (HCC) is the world’s sixth most common cancer, with increasing incidence. Over the years, there has been significant variation in prevalence of risk factors for HCC around the world, such as control of viral hepatitis in developing countries and fatty liver disease in the developed world. Trends in these risk factors are related to changing epidemiology of HCC [1]. HCC accounts for more than 500 to 600 thousand deaths per year worldwide [2]. 

In recent decades, significant progress has been made in understanding the complex role of autophagy (i.e., the cellular process that eliminates molecules and subcellular elements via lysosome-mediated degradation) in cancer regulation, including in HCC. Despite controversy, it is generally accepted that this cellular process can be overactivated, dysregulated, or suppressed in cancer cells, and its roles in regulating cancer are dependent on different stages of tumorigenesis [3]. In particular, autophagy is recognized to prevent chronic cellular damage and delay cancer-initiation cells in early stages through elimination of toxic unfolded proteins, oncogenic protein substrates, and damaged organelles, and to contribute to immunosurveillance maintenance [4,5]. Conversely, after malignant cells are established in advanced stages, enhancement of autophagy promotes tumor-cell survival and growth [6,7].

Herbal medicine uses natural ingredients that can serve a variety of treatment purposes, including for cancer [8]. The genus *Alocasia* (family: Araceae) consists of more than 100 species, including herbaceous, perennial, and large plants, that grow in subtropical and tropical regions around the world, such as Asia, the Western Pacific, and Eastern Australia [9]. Distinct parts of plants in the *Alocasia* species are commonly used in traditional medicine to treat coughs, toothache, malaria, and abscesses, and as nyctalopic agents for people who are unable to see clearly in low light [10]. Moreover, several studies have demonstrated potential of *Alocasia* plants to serve as anticancer agents. In particular, it was reported that the butanol extract of *A. cucullata* demonstrates a potent antitumor effect both in vitro and in vivo via antiproliferation of G0/G1 arrest and cell pro-apoptosis, including the PI-3 K/Akt pathway, ERK activity, stimulated cytochrome C release, and caspase 3/7 activity, along with an increase in Bax/Bcl-2 ratio [11]. Additionally, the aqueous extract of *A. macrorrhiza* exhibits anticancer action against hepatic cancer through suppression of proliferation and influences apoptosis in human hepatocellular carcinoma cells, both in vitro and in vivo [12]. Moreover, it has been shown that a 50% ethanolic extract of *A. cucullata* displays in both in vitro and in vivo anti-malignant melanoma activity through alteration of the phosphatase and tensin homolog/phosphoinositide 3-kinase/AKT pathways [13]. Additionally, the water extract of *A. cucullata* roots shows an antitumor effect via activation of antitumor immunity. In terms of its mode of action, the extract strongly stimulated THP-1 differentiation into macrophage-like cells.

*A. gigantea* is one important species of the *Alocasia* genus, commonly known as “Giant Elephant Ear” or “Giant Taro”, and it is widespread in tropical zones, such as Southeast Asia [14]. To the best of our knowledge, phytochemical profiles and bioactive properties of this species remain unknown. Taking this into consideration, this study intends to elucidate phytochemical constituents of a defatted methanolic extract of *A. gigantea* leaves (DefMeOH-E) and simultaneously evaluate *A. gigantea*’s in vivo anticancer activity, particularly via inducement of autophagy in hepatocellular carcinoma.

## 2. Results and Discussion

### 2.1. Phenolic Profile of A. gigantea Defatted Methanolic Extract

**Figure 1:**
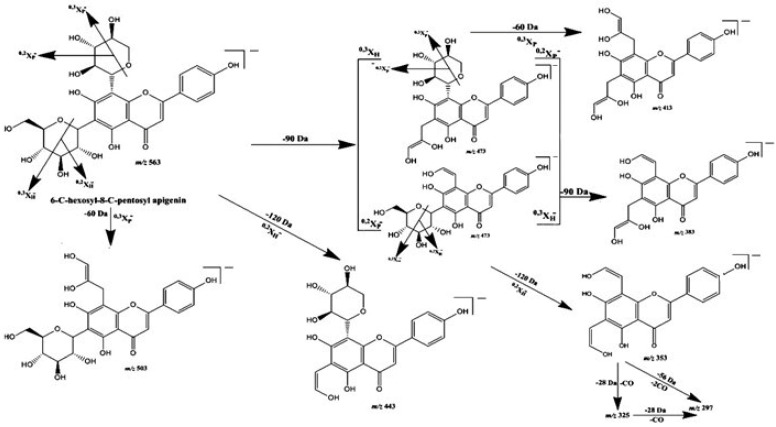
Proposed fragmentation pattern of 6-*C*-hexosyl-8-*C*-pentosyl apigenin in negative ion mode.

The total phenolic compounds in the defatted methanol extract of *A. gigantea* represented 293.02 mg GAE/g dry extract, which, based on the UHPLC–DAD–ESI–MS/MS analysis, was identified as di-*C*-glycosyl flavones (Figure S1, Table 1), particularly from apigenin (peaks 3–7; UV_max_ 271, 334). These compounds were detected in the MS spectrum as [M-H]^−^ at *m*/*z* 563 and, as represented in Figure 1, showed fragment ions that corresponded to aglycone apigenin plus the residues of the sugar, i.e., at *m*/*z* 383 [Apigenin + 113]^–^ and at *m*/*z* 353 [Apigenin + 83]^–^, as well as those resultant from sugar breakage (i.e., at *m*/*z* 545 [(M-H)-18]^−^, *m*/*z* 503 [(M-H)-60]^−^, *m*/*z* 473 [(M-H)-90]^−^, and *m*/*z* 443[(M-H)-120]^−^) assigned to 6-*C*-pentosyl-8-*C*-hexosyl apigenin or 6-*C*-hexosyl-8-*C*-pentosyl apigenin derivatives [15,16]. As *C*-6-isomers are easier to fragment than are *C*-8-isomers [17], and the ion [(M-H)-60]^−^ (characteristic of pentose derivatives) was much less abundant in compounds eluted in peaks 3, 5, and 7 compared to its abundance in compounds from peaks 4, 6, and 8, this data also allowed us to conclude that 6-*C*-hexosyl-8-*C*-pentosyl apigenin derivatives were the predominant isomers in DefMeOH-E. The most common sugars involved in the glycosylated flavonoids are the hexoses glucose and galactose, the deoxyhexose rhamnose, and the pentoses arabinose and xylose [18]. In addition to di-*C*-glycosyl apigenin isomers, two 6-*C*-hexosyl-8-*C*-pentosyl luteolin derivatives were detected as minor components in DefMeOH-E (peaks 1 and 2; UV_max_ 271, 346–348; [M-H]^−^ at *m*/*z* 579→561, 519, 489, 459, 399, 369). Metabolite profiling of the *Colocasia esculenta* species, which belongs to its closest genus *Colocasia*, via HPLC–DAD–ESI/MS led to identification of 30 glycosylated flavonoids—among them flavone mono-*C*-glycosides and flavone di-*C*-glycosides—in which luteolin-6-*C*-hexoside was the predominant identified compound. The obtained results are in full agreement with our current findings [17].

### 2.2. Anticancer Potential of A. gigantea Leaf Defatted Methanolic Extract

#### 2.2.1. In Vitro Cytotoxic Activity on HCC Cell Line

**Figure 2:**
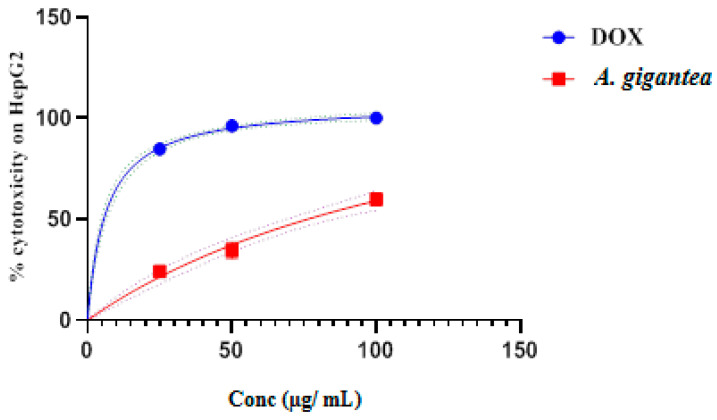
*In vitro* cytotoxic activity of *A. gigantea* leaf defatted methanolic extract at different concentrations (100, 50, and 25 µg/mL) in comparison to standard drug doxorubicin.

Anticancer activity of DefMeOH-E was tested first on a hepatic cancer line, HepG2cell, using a crystal violet assay to determine cells’ vitality [19]. As shown in Figure 2, exposure of those cells to increasing concentrations of the extract (in the range of 25 and 100 µg/mL) enhanced cytotoxic activity to an IC_50_ value of 76.33 µg/mL. A previous study on another species of *Alocasia*, namely *A. macrorrhiza*, exhibited proliferation inhibition and apoptosis effects on human hepatocellular carcinoma cells in vitro as well as inhibiting hepatoma growth with an IC_50_ value of 414 μg/mL [12]. This result suggests that DefMeOH-E of *A. gigantea* origin has potential anticancer activity against HCC. 

#### 2.2.2. In Vivo Acute Toxicity

The first step in determining in vivo toxicity of a plant extract is to conduct an acute oral toxicity test [20]. Therefore, animals were fed with DefMeOH-E at a dose of 2000 mg/kg after a fasting period of 12 h and only allowed to drink water, followed by monitoring of clinical and behavioral signs in the first 4 h, at 72 h, and after 7 days. No mortality or clinical signs of toxicity were observed. Additionally, after 7 days of observation, no mortality was detected. Based on these results, DefMeOH-E was considered non-toxic. For chemical substances and mixtures, the globally harmonized classification system (GHS) of the Organization for Economic Cooperation (OECD) classifies substances with LD_50_ > 2–5 g/kg as unclassified or category 5. This implies that the plant’s oral LD_50_ of 2–5 g/kg may be safe [21]. 

#### 2.2.3. In Vivo Body and Liver Weight and Biochemical Parameters

Animal models of HCC include xenograft models, genetically modified mouse models, and chemically induced models [22]. In the past few years, diethylnitrosamine (DEN) has been widely applied as an “initiating agent” within a myriad of protocols in mice and rats for HCC development [23,24]. It is known to cause changes in enzymes required in DNA repair replication and is regularly utilized as a cancer-causing agent to prompt liver carcinogenesis in mouse models. In this case, it was given as an intraperitoneal injection: a common technique that safely delivers a substance into the peritoneal cavity but can induce high stress in animals [24]. Overall, 48 animals were divided into four groups (12 mice/group), and this study lasted 12 weeks, with normal conditions set for Group I (Gp-I), in which mice were intraperitoneally administered a saline solution once a week, while Group III (Gp-III, *A. gigantea*) was orally administered the plant extract twice a week at 150 mg/kg body weight (BW). In turn, animals in Group II (Gp-II, DEN) were HCC-induced by an intraperitoneal injection of DEN at 3.5 µL/mg BW twice a week, and Group IV (Gp-IV, DEN/*A. gigantea*) was also given DEN twice a week for 12 weeks, combined with an oral treatment of DefMeOH-E at 150 mg/kg in the final four consecutive weeks. 

**Figure 3:**
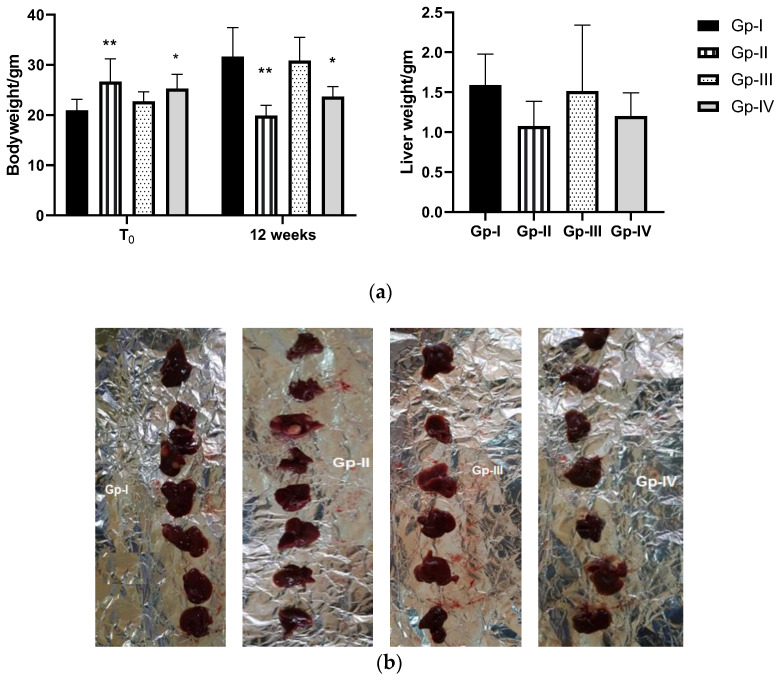
(**a**) Effect of *A. gigantea* leaf defatted methanolic extract on body weight (at initial time and 12 weeks) and liver weight (at 12 weeks) of different mouse groups. Gp-I: Group I, normal group; Gp-II: Group II, treated with DEN; Gp-III: Group III, treated with *A. gigantea*; Gp-IV: Group IV, treated with DEN combined with plant extract. Data represent the mean ± SEM. * *p* < 0.05, ** *p* < 0.01 indicate significant differences compared to Gp-I. (**b**) This photo represents liver mass between different groups.

As observed in Figure 3, body weight (BW) of animals just before termination (12 weeks) was different from that registered at the initial point (T_0_). It increased in Gp-I and Gp-III, while the opposite trend was noticed in Gp-II, which registered 25.45% weight loss (*p* = 0.0012). Notably, this was reversed in part by the *A. gigantea* treatment, as animals in Gp-IV registered a lower decrement in BW compared to those of Gp-II. Liver weight (LW) just before termination was nearly the same in Gp-I (BW = 31.65 ± 5.76; LW = 1.59 ± 0.39) and Gp-III (BW = 30.85 ± 4.64; LW = 1.51 ± 0.83). A decrease in LW was detected in Gp-II (BW = 19.9 ± 2.03; LW = 1.075 ± 0.31), which was related to BW loss in that group. Gp-IV showed a slight increase in LW compared to Gp-II (BW = 22.68 ± 1.97; LW = 1.2 ± 0.29).

The liver function was evaluated through examination of the liver enzymes alkaline phosphatase (ALP), alanine transaminase (ALT), and aspartate aminotransferase (AST), as well as the liver waste product total bilirubin (TBILR). Results in Table 2 show that all tested parameters in Gp-II (DEN) were significantly higher when compared to Gp-I (normal group) (*p* < 0.0001). In addition, treatment with DefMeOH-E in Gp-IV caused a remarkable decrease in all liver function parameters compared with the HCC group (Gp-II) (*p* < 0.0001). Overall, these results indicate that serum transaminase ALT, AST, and TBILR activities increased significantly after DEN induction. Overproduction of these proteins in tumor cells is caused by DEN-induced changes in permeability of the cell membrane, resulting in protein leakage into serum [25,26]. In turn, treatment with *A. gigantea* extract in Gp-IV caused reduction in elevated activities of these proteins, which may be due to effective compounds in *A. gigantea* maintaining parenchymal cell recovery in the liver, leading to a decrease in enzymatic leakage [27].

The majority of *Alocasia* species research focuses on hepatoprotection, mostly performed with *Alocasia indica* (Roxb.), showing that the ethanolic *A. indica* leaf extract reduces hepatotoxicity [28]. The hepatoprotective effect of the *Alocasia indica* tuber extract has also been demonstrated on albino Wistar rats with CCl_4_-induced liver injury, particularly in ethanolic extract given at 200 mg/kg for 7 days [29]. In addition, *Alocasia macrorrhiza* was also employed in trials as a hepatoprotective agent, reducing leakage of AST, ALT, and ALP in rats with CCl_4_- and Tylenol-induced liver injury [28]. *Alocasia macrorrhiza* also has an anticancer effect on various cell lines, inhibiting growth of hepatoma in vivo [12]. 

#### 2.2.4. Histopathological Examinations

**Figure 4:**
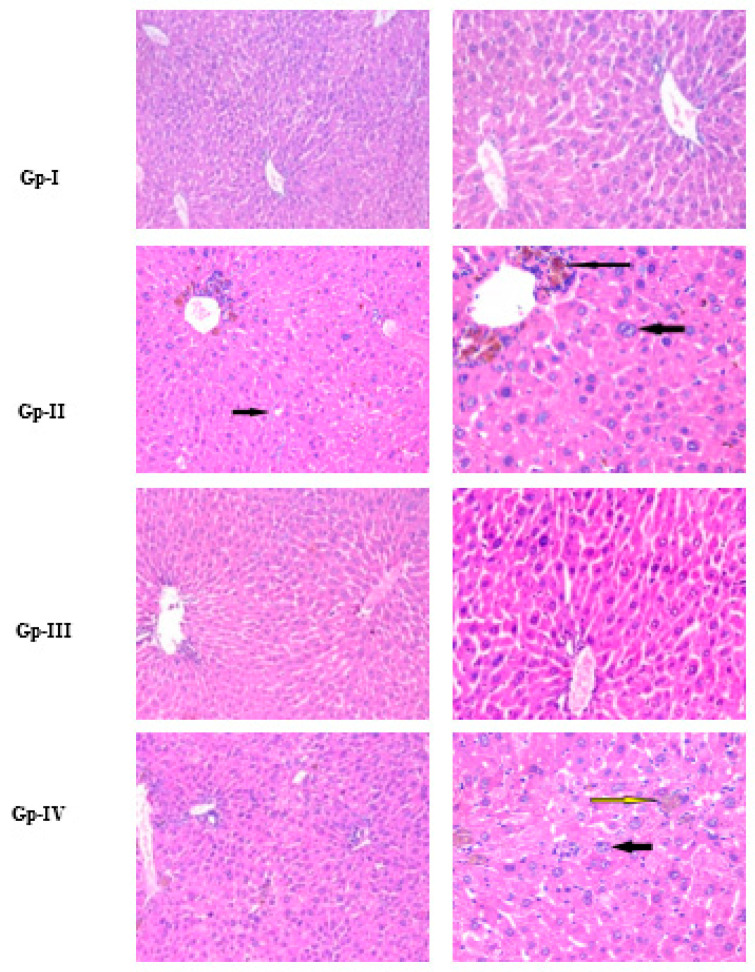
Histopathology using hematoxylin and eosin (H&E) staining of the liver, isolated from each animal group used in this study. For each group, the photo on the right side is a zoom-in taken from the photo on the left. Gp-I: Group I, normal group; Gp-II: Group II, treated with diethylnitrosamine; Gp-III: Group III, treated with *A. gigantea*; Gp-IV: Group IV, treated with diethylnitrosamine combined with plant extract. Thick and thin black arrows in Gp-II indicate HCC with focal acinar formation and bizarre-shaped hyperchromatic nuclei and focal cholestasis, respectively. Thin yellow and black arrows in Gp-IV indicate focal hepatocellular dysplasia with focal cholestasis and few apoptotic figures, respectively.

Histopathological images are represented in Figure 4. Hepatocytes with granular cytoplasm that occupied the acidophilic stain, as well as centrally located nuclei, were seen in the histology of both the Gp-I (normal) and the Gp-III (*A. gigantea*) group. With hematoxylin and eosin (H&E) staining, the central vein and bile ducts could be seen. In the histology of Gp-II (DEN), distorted architecture, focal HCC, and dysplasia were seen, as well as areas of necrosis, cholestasis, bile duct proliferation, and lymphatic dilatation. However, in Gp-IV (DEN/*A. gigantea*), hepatic architecture was more preserved, with minor hepatocytic changes but scattered foci of hepatocytic apoptosis. On the other hand, Gp-III (*A. gigantea*) showed preserved hepatic lobular architecture with no histopathological changes. Dysplastic foci are homogeneous lesions that can be distinguished from the surrounding liver tissue by their distinctive morphology, cytoplasmatic staining, nuclear size, and cellular atypia. Due to their elevated proliferation index and poor apoptosis rate, they are additionally regarded as premalignant lesions [30]. Another histological HCC marker is cholestasis, which is almost always extracellular, localized at the biliary pole of the tumor hepatocytes [31].

Despite the absence of studies with *A. gigantea*, a preservative effect on normal morphology, in addition to antitumor properties against tumoral hepatic cells, has been demonstrated for other *Alocasia* species: in particular, hepatotoxic protective effects of *A*. *indica* tuber extract in alcohol-intoxicated rats, where recovery from ethanol-induced liver damage was observed, with fewer micro-vesicular steatoses, hepatocytes necrosis features, and absence of fat droplets [29]. *A. indica* leaf extract has also been shown to reduce inflammation, degenerative changes, and steatosis in liver tissue treated with CCl_4_ and paracetamol [28]. 

#### 2.2.5. In Vivo Antitumoral Effects through Induction of Autophagy

One of the characteristics of cancer is alteration in cell death; these cells are under survival pressure. They alter as a result of failure of apoptosis, which causes genetic harm [32]. Mutational and expressional alterations of apoptosis genes such as Fas and caspase are abundant in different types of human cancer [33,34]. However, compared to apoptosis, information on autophagy genes and their function in cancer is significantly limited [6,35]. With this in mind, this study focused on autophagy in HCC, which is a topic under debate. 

Table 3 summarizes the results of the relative expression of serum tumor necrosis factor-alpha (TNF-α) and alfa-fetoprotein (AFP), which are central inflammatory and tumor markers, respectively. Overall, the gathered data indicate that treatment with DefMeOH-E did not induce inflammatory or tumorigenic effects in animals (Gp-III). Conversely, injection of DEN in Gp-II caused a large increase in animals’ serum levels of TNF-α and AFP genes, which was significantly reversed by the *A. gigantea* treatment in Gp-IV (DEN/*A. gigantea*). Accordingly, a previous study concluded that combined use of TNF-α and AFP increases sensitivity and specificity for early diagnosis of HCC, as their increased expression is related to HCC [36]. 

Generally, the process of autophagy entails formation of a double-membrane vesicle that encloses cytoplasm, abnormal proteins, long-lived proteins, and organelles before joining with lysosomes for breakdown. The molecular mechanism of autophagy is complex and involves distinct autophagy-related (Atg) proteins. In this study, the effect of *A. gigantea* extract on autophagy was evaluated through monitoring of autophagy gene markers involved in the initiation step of autophagy: namely adenosine monophosphate-activated protein kinase (AMPK) and mammalian target of rapamycin complex 1 (mTORC1) complexes, the Beclin-1-class III phosphatidylinositol 3-kinase (PI3K) complex (which mediates nucleation of the phagophore to form autophagosome), and LC3 (considered the mature autophagosome marker) [37].

**Figure 5:**
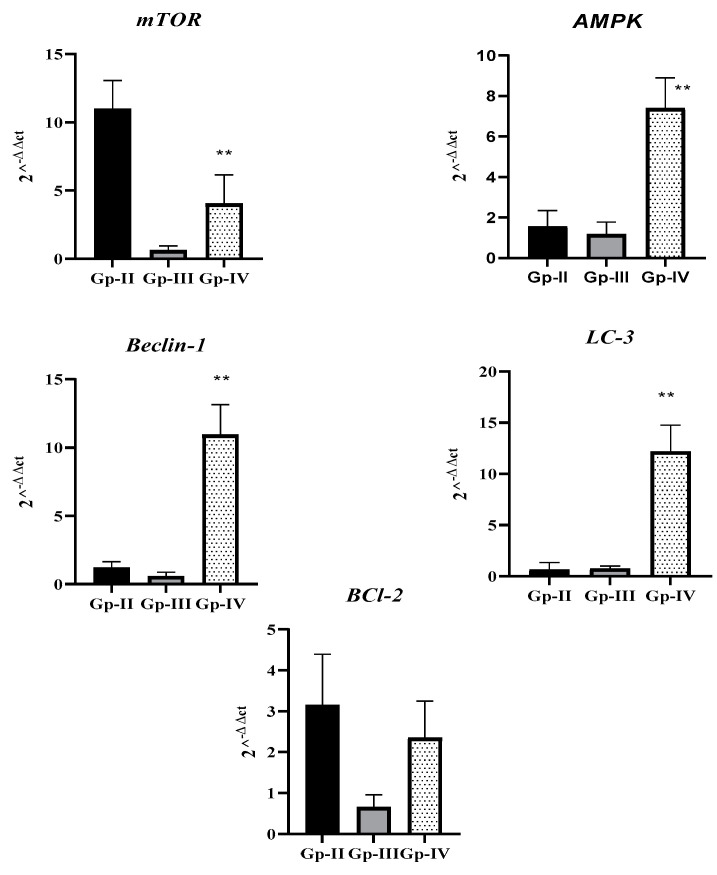
Relative quantification (RQ = 2^−ΔΔct) of tumor marker BCl-2 and autophagy markers (mTOR, AMPK, Beclin-1, and LC-3) in different animal groups, in relation to Gp-II (DEN group). Gp-II: Group II, treated with diethylnitrosamine; Gp-III: Group III, treated with *A. gigantea*; Gp-IV: Group IV, treated with diethylnitrosamine combined with plant extract. Data represent the mean ± SEM. ** *p* < 0.01 indicates significant differences from the DEN group.

As represented in Figure 5, tumor marker BCl-2 was higher in Gp-II than in the other groups. In addition, mTOR, an autophagy suppressor, was significantly lower in Gp-IV when compared to Gp-II (*p* < 0.0001). In accordance, autophagy markers (AMPK, Beclin-1, and LC-3) also displayed markedly increased expression in Gp-IV (*p* < 0.0001). Taken together, the data collected indicate that combined treatment of DEN with *A. gigantea* extract promoted autophagy and decreased tumor markers in animals.

Previous studies proved that polyphenolic compounds displayed anti-HCC effects through autophagy via interference with canonical (Beclin-1-dependent) and non-canonical (Beclin-1-independent) pathways [38]. Moreover, apigenin has been shown to exhibit anti-cancer properties in various types of cancer, including breast, liver, prostate, lung, and colon cancer, in addition to anti-inflammatory and antioxidant effects [39]. The anti-HCC effect of apigenin through down-regulation of the NF-κB pathway has been shown [40]. Considering the results from our study, it is possible to suggest that apigenin derivatives, i.e., the main phenolic compounds in the *A. gigantea* extract, may play a relevant role in apigenin’s antitumoral effect; however, this hypothesis must be further consolidated. 

## 3. Materials and Methods

### 3.1. Plant Material

*Alocasia gigantea* leaves were collected after permission and in compliance with relevant international guidelines and legislation from Experimental Plants Station, Faculty of Pharmacy, Cairo University, Giza, Egypt, during April 2020. Identification and authentication of the plant material were achieved by Dr. Tearse Labib, consultant of taxonomy at the Ministry of Agriculture and former director of El-Orman Botanical Garden, Giza, Egypt. A voucher specimen (No. A.g/l/2020) is kept in the herbarium of the Medicinal Chemistry Department at Theodor Bilharz Research Institute. 

### 3.2. Extraction and Defatting 

Dry powdered leaves of *A. gigantea* (1100 g) were extracted four times with methanol via maceration (4 L) at room temperature. The combined extracts were filtered and evaporated under vacuum using a rotatory evaporator (Buchi, Flawil, Switzerland) at 40 ± 2 °C to afford a methanol extract of 120.25 g (10.93%). The dried methanol extract was defatted using petroleum ether (60–80 °C), followed by dichloromethane in order to remove undesirable compounds [41], affording 30.87 g (2.81%), 11.29 g (1.03%), and 76.09 g (6.92%), respectively, for petroleum ether, dichloromethane, and defatted methanol extracts. Next, the defatted methanol extract (DefMeOH-E) was stored for further chemical and biological investigations. 

### 3.3. Determination of Total Phenolic Content (TPC)

Total phenolic content of each extract was determined using the Folin–Ciocalteu reagent according to the reported procedure of Prior et al. (2019), with gallic acid as a standard [42]. The reaction mixture was composed of 50 μL extract (500 μg/mL), 250 μL Folin–Ciocalteu reagent, and 0.75 mL sodium carbonate (20%). The mixture was shaken, and completed to 5 mL using distilled water. The mixture was allowed to stand for 2 h; then absorbance was measured at 765 nm using a spectrophotometer (UV-vis; Milton Roy 601, Co., Houston, TX, USA). All determinations were carried out in triplicate. Total phenolic content was expressed as mg gallic acid equivalent (GAE) per g extract. 

### 3.4. UHPLC–DAD–ESI–MS/MS Analysis

This analysis was performed on an Ultimate 3000 (Dionex Co., San Jose, CA, USA) apparatus equipped with an ultimate 3000 Diode Array Detector (Dionex Co., USA) and coupled to a mass spectrometer, following the general procedure previously described [43]. The chromatographic apparatus consisted of an autosampler/injector, a binary pump, a column compartment and an ultimate 3000 Diode Array Detector (Dionex Co., San Jose, CA, USA), coupled to a Thermo LTQ XL (Thermo Scientific, San Jose, CA, USA) ion trap mass spectrometer equipped with an ESI source. The LC separation was carried out in a Hypersil Gold (ThermoScientific, San Jose, CA, USA) C_18_ column (100 mm length; 2.1 mm i.d.; 1.9 µm particle diameter; end-capped) maintained at 30 C and a binary solvent system composed of (A) acetonitrile and (B) 0.1% formic acid (*v*/*v*). The solvent gradient started with 5–40% of solvent (A) over 14.72 min, at 40–100% over 1.91 min and remaining at 100% for 2.19 more min before returning to initial conditions. The flow rate was 0.2 mL/min, and UV-vis spectral data for all peaks were accumulated in the range of 200–700 nm while chromatographic profiles were recorded at 280 nm. Control and data acquisition of MS were carried out with the Thermo Xcalibur Qual Browser data system (ThermoScientific, San Jose, CA, USA). Nitrogen above 99% purity was used, and the gas pressure was 520 kPa (75 psi). The instrument was operated in negative mode, with the ESI needle voltage set at 5.00 kV and an ESI capillary temperature of 275 °C. The full scan covered the mass range from *m*/*z* 100 to 2000. CID–MS/MS experiments were performed for precursor ions, using helium as the collision gas, with a collision energy of 25–35 arbitrary units. 

### 3.5. In-Vitro Study on HCC Cell Line

The Department of Cell Culture (Vacsera, Egypt) provided the HepG2cell line. The cells were cultured in a PYR-free 1640 RPMI medium (Thermo Fisher Scientific). The medium was made up of 10% FBS, 1% HEPES, and 1% antibiotic/antimycotic combination (LONZA). Different quantities (500, 250, and 125 g/mL) of the *A. gigantea* extract and doxorubicin (DOX) (as a standard drug) were applied following attachment of the cells (7000 cells/well) on 96 tissue culture plates. The plates were then incubated at 37 °C in 5% CO_2_ for 24 h. Cell viability was detected using a crystal violet assay following the general procedure previously described [19].

### 3.6. Animals

All animal experiments were carried out under Institutional Ethical Committee rules for care and use of experimental animals, authorized by Theodor Bilharz Research Institute’s Animal Ethics Committee in Giza, Egypt (PT (583)/FWA 00010609). Theodor Bilharz Research Institute’s animal house provided a total of 48 male Swiss albino mice (6–7 weeks old) weighing 23 ± 5 g. The animals were given a week to acclimate. Throughout the experiment, all animals were kept in standardized hygienic conditions, including a temperature of 21–22 °C, a humidity of 55%, a standard 12 h light-dark cycle, and food and water accessibility.

### 3.7. Assessment of A. gigantea Acute Toxicity

The first step in determining toxicity of a plant extract is to conduct an acute oral toxicity test. The animals were starved for 12 h and only allowed to drink water. They were then weighed after the fasting period, and a test extract was given orally at a dose of 2000 mg/kg. Food was withheld from the animals for 2 h after the test extract was administered. In the first instance, mortality; clinical signs, such as changes in the skin, fur, eyes, and mucous membranes; and behavioral signs, as diarrhea, lethargy, sleep, or tremors were tracked for the first 4 h, then at 72 h and at 7 days after the test extract was administered [44,45].

### 3.8. In Vivo Experimental Design

To reach HCC, diethylnitrosamine (DEN) was used as an inducer. Intraperitoneal injection is a common technique that safely delivers a substance into the peritoneal cavity but can induce high stress in animals. Therefore, we depended on it to deliver HCC [46]. Mice were divided into 4 groups (12 mice/group) that were administered for 12 weeks. Gp-I (normal), a control group, was given a saline solution intraperitoneally (i.p.) (3.5 µL/mg BW); Gp-II (positive) was given diethylnitrosamine (DEN) twice a week i.p. (3.5 µL/mg BW); Gp-III (*A. gigantea*) was given plant extract (150 mg/kg BW) orally twice a week; and Gp-IV (DEN/*A. gigantea*) was also given DEN twice a week i.p. (3.5 µL/mg BW). At the end of 8 weeks, Gp-IV was treated with plant extract (150 mg/kg) orally twice a week for 4 consecutive weeks in combination with DEN.

After the required time was reached, scarification was performed. Euthanasia was chosen to minimize animal pain and distress consistent with the needs of the research protocol. Euthanasia was performed via inhalation of CO_2_ from a pressurized tank in a rodent cage that contained up to 5 adult mice, followed by cervical dislocation and decapitation. CO_2_ (30–70% displacement per minute depending on cage size) gas flow was slow and neither hissed nor overpowered and frightened the mice [47].

### 3.9. Body and Liver Weight and Biochemical Parameters 

Body weight for each group was registered at the beginning of this study and before termination. Liver weight for each group was detected after scarification. The mice fasted overnight after the last treatment. Collected blood samples were centrifuged at 2000 rpm for 10 min. A liver function test was monitored using a serum aspartate aminotransferase (AST) and alanine transaminase (ALT) kit (Sclavo Diagnostics Internationals), an alkaline phosphatase (ALP) kit (N.S. BIO-TEC), and a total direct bilirubin (TBILR) kit (Sclavo Diagnostics Internationals).

### 3.10. Histopathological Examinations

The isolated livers were fixed in 10% buffered formalin. The liver was routinely processed into paraffin blocks. On positively charged glass slides, 4–5 µm thick sections were cut. Sections were then stained with hematoxylin and eosin (H&E) for light microscopic histopathological examination of hepatic architecture, inflammation, dysplasia, and carcinogenesis. The Masson trichrome stain was used to assess tissue fibrosis. Liver histology of different groups was compared using a Zeiss Axio microscope, and photos were taken with the attached digital Mrc5 camera (Zeiss).

### 3.11. Inflammatory, Tumoral, and Autophagy Markers

A commercially available kit was used to isolate total RNA from serum and liver tissues (Biovision, Inc., Milpitas, CA, USA). To detect gene expression of tumor and autophagy markers, quantitative PCR (qPCR) was performed using isolated RNA (1 µg), a cDNA synthesis kit (Biovision, Inc.), and SYBR green master mix (Thermo Fisher Scientific). Each primer’s sequence was designed as shown in Table 4. The mean with SD of each detected marker in each group was used to describe relative expression, using the following equation: RQ = 2^−ΔΔct.

### 3.12. Statistical Analysis

Data in treatment groups were presented as mean with SD, and statistical analysis was performed using GraphPad Prism 8 (San Diego, CA, USA). One-way or two-way ANOVA was followed by a post-hoc Tukey multiple comparison test. *p* < 0.05 was determined to be statistically significant.

## 4. Conclusions

Chemical characterization of the defatted methanol extract of *A. gigantea* using UHPLC–DAD–ESI–MS/MS analysis led to identification of eight di-*C*-glycosyl flavone isomers of apigenin and luteolin. Moreover, the conducted study allowed conclusion that the extract of *A. gigantea* has potential anti-HCC effects via modulation of the autophagy pathway. The outcomes reached give an initial visualization of the operative effect of some compounds, paving the way to extensive study on isolation and activity of individual compounds.

## Figures and Tables

**Table 1:** Phenolic compounds detected in *A. gigantea* leaves defatted methanolic extract via UHPLC–DAD–ESI–MS/MS analysis.

Peak	RT (min)	λmax (nm)	*m*/*z*	ESI–MS^2^	Compound
1	9.4	271, 348	579	561, 519, 489, 459, 399, 369	6-*C*-hexosyl-8-*C*-pentosyl luteolin (isom **1**)
2	9.7	271, 346	579	561, 519, 489, 459, 399, 369	6-*C*-hexosyl-8-*C*-pentosyl luteolin (isom **2**)
3	10.1	271, 333	563	545, 503, 473, 443, 413, 383 353	6-*C*-hexosyl-8-*C*-pentosyl apigenin (isom **1**)
4	10.4	271, 333	563	545, 503, 473, 443, 383, 353	8-*C*-hexosyl-6-*C*-pentosyl apigenin (isom **1**)
5	10.7	271, 333	563	545, 503, 473, 443, 383, 353	6-*C*-hexosyl-8-*C*-pentosyl apigenin (isom **2**)
6	11.0	271, 333	563	545, 503, 473, 443, 383, 353	8-*C*-hexosyl-6-*C*-pentosyl apigenin (isom **2**)
7	10.4	271, 333	563	545, 503, 473, 443, 383, 353	6-*C*-hexosyl-8-*C*-pentosyl apigenin (isom **3**)
8	11.9	271, 333	563	545, 503, 473, 443, 383, 353	8-*C*-hexosyl-6-*C*-pentosyl apigenin (isom **3**)

Isom: Isomer.

**Table 2:** Serum-liver-function parameters for each animal group used in this study.

Liver Markers	Gp-I	Gp-II	Gp-III	Gp-IV
TBILR	0.22 ± 0.18	1.27 ± 0.12 ^c^	0.16 ± 0.07	0.60 ± 0.14 ^b^
ALP	8.95 ± 1.75	24.99 ± 2.4 ^c^	12.39 ± 2.1 ^c^	14.66 ± 1.22 ^c^
ALT	7.45 ± 1.86	35.83 ± 3.7 ^c^	10.25 ± 1.8 ^a^	21.88 ± 5.89 ^c^
AST	112.51 ± 8.1	148.62 ± 5.4 ^c^	93.99 ± 22.59 ^a^	116.52 ± 27.25

Results are expressed in mg/dL (for total direct bilirubin) and international unit per liter (IU/L) for alkaline phosphatase (ALP), alanine transaminase (ALT), and aspartate aminotransferase (AST). Gp-I: Group I, normal group; Gp-II: Group II, treated with diethylnitrosamine; Gp-III: Group III, treated with *A. gigantea*; Gp-IV: Group IV, treated with diethylnitrosamine combined with plant extract. In each line, different letters represent significant differences compared to Gp-I: a—*p* < 0.05, b—*p* < 0.01, c—*p* < 0.001.

**Table 3:** Effect of *A. gigantea* leaf defatted methanolic extract on TNF-α and AFP relative expression in each animal group.

Serum Markers	Gp-I	Gp-II	Gp-III	Gp-IV
TNF-α	0.068 ± 0.024	322.24 ± 11.2 ^c^	4.25 ± 2.1 ^c^	194.54 ± 15.92 ^c^
AFP	0.98 ± 0.299	4.48 ± 1.15 ^c^	0.65 ± 0.25 ^a^	2.9 ± 0.97 ^c^

Results are expressed in relative quantification (RQ) for TNF-α (tumor necrosis factor-alpha) and AFP (alpha-fetoprotein). Gp-I: Group I, normal group; Gp-II: Group II, treated with diethylnitrosamine; Gp-III: Group III, treated with *A. gigantea*; Gp-IV: Group IV, treated with diethylnitrosamine combined with plant extract. In each line, different letters represent significant differences compared to Gp-I: a—*p* < 0.05, b—*p* < 0.01, c—*p* < 0.001.

**Table 4:** Primer sequences for gene expression analysis using qPCR.

Gene	Primer Sequence	Reference
β-actin	Sense: GGGAATGGGTCAGAAGGACT	[48]
	Antisense: CTTCTCCATGTCGTCCCAGT	
BCl-2	Sense: ATGCCTTTGTGGAACTATATGGC	[49]
	Antisense: GGTATGCACCCAGAGTGATGC	
mTOR	Sense: GGCCAAAAGGCAGGTGGCT	This study
	Antisense: ATGTTCACTTTGTGCTTGTA	
AMPK	Sense: GGAGAATAATGAATGAAGCC	This study
	Antisense: CACCTTGGTGTTTGGATTTC	
Beclin-1	Sense: GAGAGACCCAGGAGGAAG	This study
	Antisense: GGCCCGACATGATGTCAA	
LC-3	Sense: CCCGGTGATCATCGAGCGCT	This study
	Antisense: GAAGGCCTGCGTGGGGTT	
AFP	Sense: CTACATTTCGCTGCGTCCAA	This study
	Antisense: CAGCCAACACATCGCTAGTC	
TNF-α	Forward: ACCCTCACACTCACAAACCA	[50]
	Reverse: GGCAGAGAGGAGGTTGACTT	

## Data Availability

Data are contained within the article.

## Supplementary Materials

The following supporting information can be downloaded at: https://www.mdpi.com/article/10.3390/molecules27238504/s1, Figure S1: UHPLC chromatogram (at 280 nm) of *A. gigantea* defatted methanolic extract. Peak numbers correspond to those in Table 1.
