# Supplementary material for: Metabolite Profiling of Alocasia gigantea Leaf Extract and Its Potential Anticancer Effect through Autophagy in Hepatocellular Carcinoma
Source: Molecules. 2022 Dec 3;27(23):8504. doi: 10.3390/molecules27238504 (PMC9740247; doi:10.3390/molecules27238504)
Supplement: Supplementary file 1 [file molecules-27-08504-s001.zip › molecules-2035792-supplementary.pdf]

Metabolite profiling of *Alocasia gigantea* leaves extract and its potential anticancer effect through autophagy in hepatocellular carcinoma

Hend Okasha[1], Tarek Aboushousha[2], Manuel A. Coimbra[3], Susana M. Cardoso[3,*] and Mosad A. Ghareeb [4,*]

[1] Department of Biochemistry and Molecular Biology, Theodor Bilharz Research Institute, Kornaish El Nile, Warrak El-Hadar, Imbaba (P.O. 30), Giza 12411, Egypt

[2] Department of Pathology, Theodor Bilharz Research Institute, Kornaish El Nile, Warrak El-Hadar, Imbaba (P.O. 30), Giza 12411, Egypt

[3] LAQV-REQUIMTE & Department of Chemistry, University of Aveiro, Aveiro 3810-193, Portugal

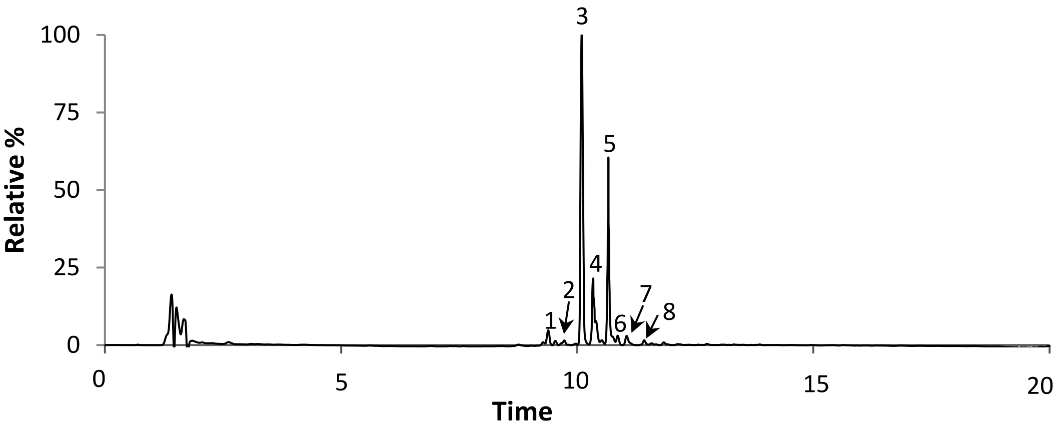

Figure S1. UHPLC- chromatogram (at 280 nm) of *A. gigantea* defatted methanolic extract in negative ion mode. Peak numbers correspond to those in Table 1.
